# Oxygen Effects on Performance of Electrically Bistable Devices Based on Hybrid Silver Sulfide Poly(*N*-vinylcarbazole) Nanocomposites

**DOI:** 10.1186/s11671-016-1289-9

**Published:** 2016-02-03

**Authors:** Jiantao Li, Aiwei Tang, Xu Li, Miao Wang, Yufeng Hu, Yanbing Hou, Feng Teng

**Affiliations:** Key Laboratory of Luminescence and Optical Information, Ministry of Education, School of Science, Beijing Jiaotong University, Beijing, 100044 China; Department of Chemistry, School of Science, Beijing Jiaotong University, Beijing, 100044 China

**Keywords:** Electrical bistability, Ag_2_S, PVK, Oxygen effects

## Abstract

An organic/inorganic bistable device is fabricated by using a simple spin-coating technique, in which the hybrid silver sulfide (Ag_2_S) poly(*N*-vinylcarbazole) (PVK) nanocomposite film is sandwiched between two electrodes. An obvious electrical hysteresis is observed in the current-voltage (*I*-*V*) curve of the device measured in the presence of different oxygen concentrations, and the magnitude of the electrical hysteresis is decreased with a decrease of the oxygen concentrations. The electrical bistability of the device exhibits a strong dependence on the oxygen concentrations, and the current variation of the OFF state is higher than that of the ON state with the gas atmosphere changing from N_2_ to air. Different theoretical models have been employed to describe the carrier transport mechanisms of the device in the OFF and ON states measured in different gas atmospheres on the basis of the experimental *I*-*V* results, and the carrier transport of the device in the ON state measured in air is very different from that measured in N_2_ and low O_2_ concentrations due to the participation of oxygen vacancies in the trapping and de-trapping processes of electrons into and out of the Ag_2_S/PVK heterointerface.

## Background

In the past few decades, organic electrically bistable devices have attracted much attention due to their potential applications in the next generation of nonvolatile memory technology [1–3]. To date, various candidates have been exploited to be used in electrically bistable devices, in which hybrid inorganic/organic nanocomposites have currently aroused broad interests due to their simple fabrication and low cost [4–6]. In general, the hybrid organic/inorganic nanocomposites are formed by dissolving colloidal inorganic nanocrystals and polymers into organic solvent to form a homogeneous phase. A typical device structure for the hybrid organic/inorganic bistable device is a single nanocrystal/polymer hybrid layer sandwiched between two electrodes, in which the hybrid layer is formed by using a simple spin-coating method. Substantial research efforts in the exploitation of inorganic nanocrystals have led to the applications of different types of inorganic nanocrystals in the fabrication of hybrid organic/inorganic bistable devices, such as II-VI group semiconductors, noble metals, and graphene and metal oxide nanocrystals [7–9]. Recently, our group reported some electrically bistable devices based on dodecanethiol-capped Cu_2_S, hybrid silver sulfide (Ag_2_S), and Ag nanocrystals blending with conjugated polymer, and obvious electrical bistability and negative differential resistance (NDR) effects were clearly observed. Although some different resistive switching mechanisms, such as charge trapping [10, 11], redox reaction [12], and metallic filament conducting [13, 14], have been brought out to elucidate the charge transport process, the resistive switching mechanism is still under debate. Therefore, there is no end to study the working mechanisms of the electrically bistable devices based on organic/inorganic nanocomposites. In our previous work, an obvious electrical bistability was observed in the hybrid organic/inorganic nanocomposites [15], but the environmental gas effects on the electrical bistability and working mechanism were not discussed. Based on our previous work, herein, the effects of environmental gas on the electrical bistability of the devices were studied, and the devices were fabricated based on hybrid organic/inorganic Ag_2_S/poly(*N*-vinylcarbazole) (PVK) nanocomposites through a simple spin-coating technique. The current-voltage (*I*-*V*) characteristics of the hybrid electrically bistable devices exhibit a strong dependence on the oxygen concentrations, in which the current of ON and OFF states changes with the measurement atmosphere variation from N_2_ to air. On the basis of the experimental results, different organic electronic models were employed to describe the carrier transport of the electrically bistable devices.

## Methods

**Fig. 1:**
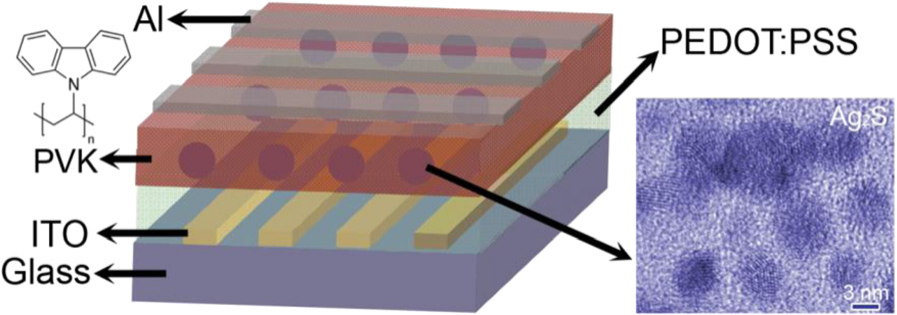
Device structure of the electronically bistable devices and the molecular structure of PVK (*left*) and HRTEM images of the as-obtained Ag_2_S nanocrystals (*right*)

The Ag_2_S nanocrystals were synthesized using a simple heating-up method based on our previous work [16], in which direct heating of the mixture containing Ag(OAc) and dodecanethiol to 200 °C for a certain time under N_2_ protection condition is performed. The hybrid electrically bistable devices based on Ag_2_S/PVK nanocomposites were fabricated on a cleaned glass substrate pre-coated with an indium-tin-oxide (ITO) anode, and then the poly(3,4-ethylenedioxythiophene):poly-(styrene-sulfonate) (PEDOT:PSS) was spin-coated onto the substrate as a buffer layer and then annealed at 150 °C for 15 min. Afterwards, the hybrid thin film was formed by using a spin-coating technique, in which the Ag_2_S nanocrystals were randomly dispersed in the PVK layer with the mass ratio of Ag_2_S to PVK being 1:1. Finally, a top Al layer with a thickness of 200 nm was deposited by thermal evaporation under the vacuum of about 1 × 10^−6^ Torr. The schematic illustration of the device structure and the molecular structure of PVK is shown in Fig. 1.

High-resolution TEM images were performed on a JEM-2010 with an acceleration voltage of 200 kV. The *I*-*V* characteristics of the devices were measured by using a Keithley Source Meter 2410 controlled by a computer. All the measurements were carried out under ambient conditions at room temperature.

## Results and Discussion

Figure 1 depicts a typical high-resolution transmission electron microscopy (HRTEM) image, and it can be seen that the morphology of the as-obtained Ag_2_S nanocrystals is quasi-spherical with a mean size less than 5 nm.

**Fig. 2:**
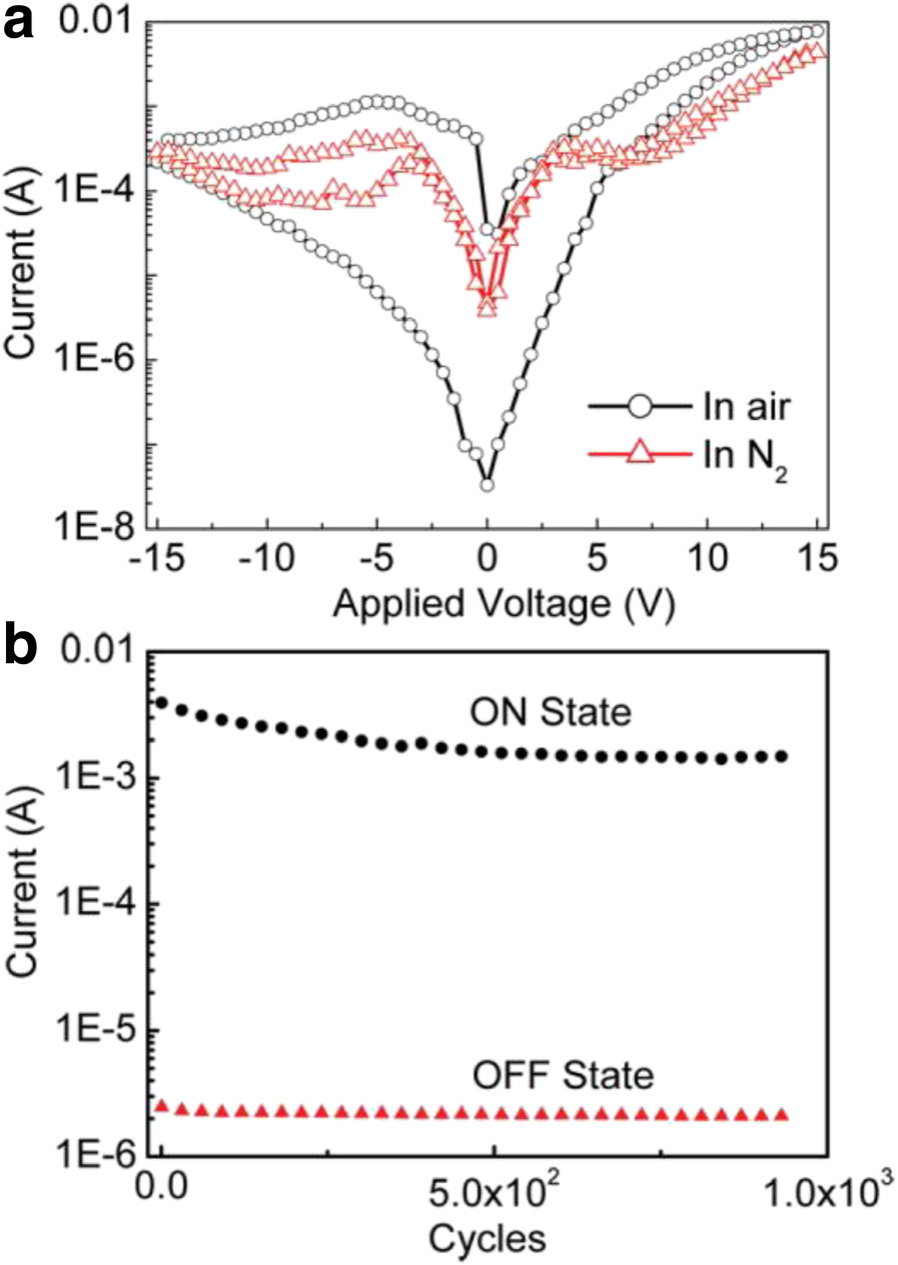
**a** *I*-*V* curve of the bistable device measured in air and N_2_ atmosphere. **b** Retention ability of the bistable devices under the sweeping voltage sequence of (10, −1, −10, 1 V) which represent the “write-read-erase-read” process

The *I*-*V* characteristics of the organic/inorganic electrically bistable devices based on hybrid Ag_2_S/PVK nanocomposites are shown in Fig. 2a, which are measured in air and in N_2_ atmosphere under the sweeping voltage from −15 to 0, 0 to15, 15 to 0, and 0 to −15 V, respectively. As shown in Fig. 2a, an obvious electrical bistability is observed when the device is measured in air, but the magnitude of the electrical hysteresis becomes smaller when it is measured in N_2_. This indicates that the measurement atmosphere has a significant effect on the electrical bistability of the hybrid bistable device. To study the retention ability of the hybrid Ag_2_S/PVK electrically bistable device, a sweeping voltage sequence of (10, −1, −10, −1 V) was applied to the device to simulate the “write-read-erase-read” process, which is measured in air. As shown in Fig. 2b, little change of OFF current and a slight degradation of ON current are observed; thus, the ON/OFF current ratio exhibits a slight degradation after operating about 1000 cycles, which indicates a reliable retention performance of the electrically bistable devices in ambient condition [17–19].

**Fig. 3:**
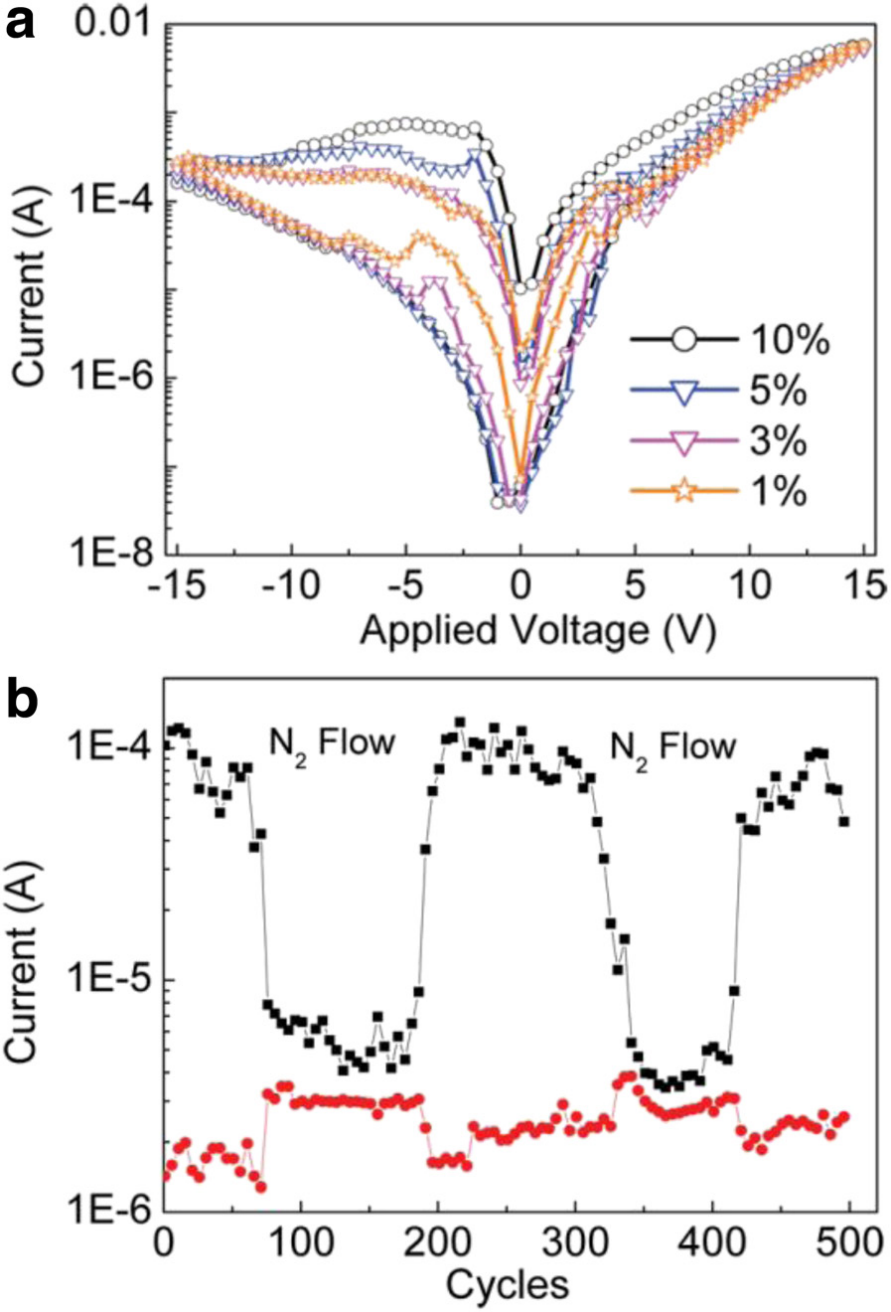
**a** *I*-*V* curves of the bistable devices measured under different O_2_ concentrations. **b** Current response of ON and OFF states under N_2_ and air flow

To further study the oxygen effects on the electrical bistability of the hybrid Ag_2_S/PVK nanocomposites, the *I*-*V* characteristics of the hybrid Ag_2_S/PVK bistable device were measured in different environmental atmospheres with different oxygen concentrations, and the corresponding *I*-*V* curves are shown in Fig. 3a. An electrical hysteresis behavior is clearly observed in the *I*-*V* curves of the electrically bistable devices measured in the atmosphere of different oxygen concentrations. The magnitude of the *I*-*V* hysteresis is enhanced with the increase of oxygen concentrations, which confirms that the oxygen concentration is important for the electrical bistability. To further study the current response of the hybrid Ag_2_S/PVK bistable device under different gaseous atmospheres, we monitor the current of ON and OFF states of the bistable device under the sweeping voltage sequence of (10, −1, −10, 1 V) by applying a sprayer to output N_2_ to the surface of the device, and the current variation of the ON and OFF states of the device in a stable period is depicted in Fig. 3b. As stated in Fig. 2b, the current of the ON and OFF states remains almost stable in the first 1000 cycles under the sweeping voltage sequence of (10, −1, −10, 1 V) measured under ambient condition. Surprisingly, it can be seen in Fig. 3b that the current of the ON state drops immediately when the device is measured under N_2_ flow, while the current of the OFF state increases slightly. Thus, the ON/OFF current ratio decreases greatly to a single digit. In contrast, the current of the ON and OFF states can be recovered when the N_2_ flow is removed. This process is operated about 500 cycles, and such a similar phenomenon is repeatable. Such a trend of the current variation is consistent with the *I*-*V* results under the different O_2_ concentrations shown in Fig. 3a. All the above experimental results suggest that the performance of hybrid organic/inorganic bistable devices based on Ag_2_S/PVK nanocomposites is very sensitive to oxygen, which may hold a potential application in gas sensor.

**Fig. 4:**
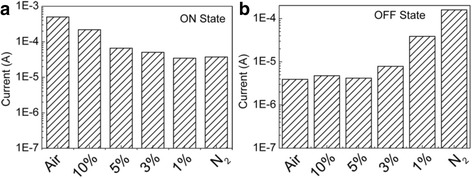
Current variation of the bistable devices measured under different atmospheres for **a** ON states and **b** OFF states

To further demonstrate the oxygen effects on the current of the ON and OFF states of the hybrid electrically bistable device, the current variation of the ON and OFF states measured under different atmospheres is given in Fig. 4a, b, respectively. As shown in Fig. 4a, it can be seen that the current of the ON state is decreased with the decrease of O_2_ concentrations, in which the highest current of the ON state can reach up to 5 × 10^−4^ A under an air atmosphere. In contrast, the current of the OFF state increases with the decrease of oxygen concentration, and the current of the OFF state is as high as 1.6 × 10^−4^ A under N_2_ atmosphere. Therefore, the oxygen concentrations have an important effect on the current variation of the ON and OFF states, which thus leads to the variation of the ON/OFF current ratios.

**Fig. 5:**
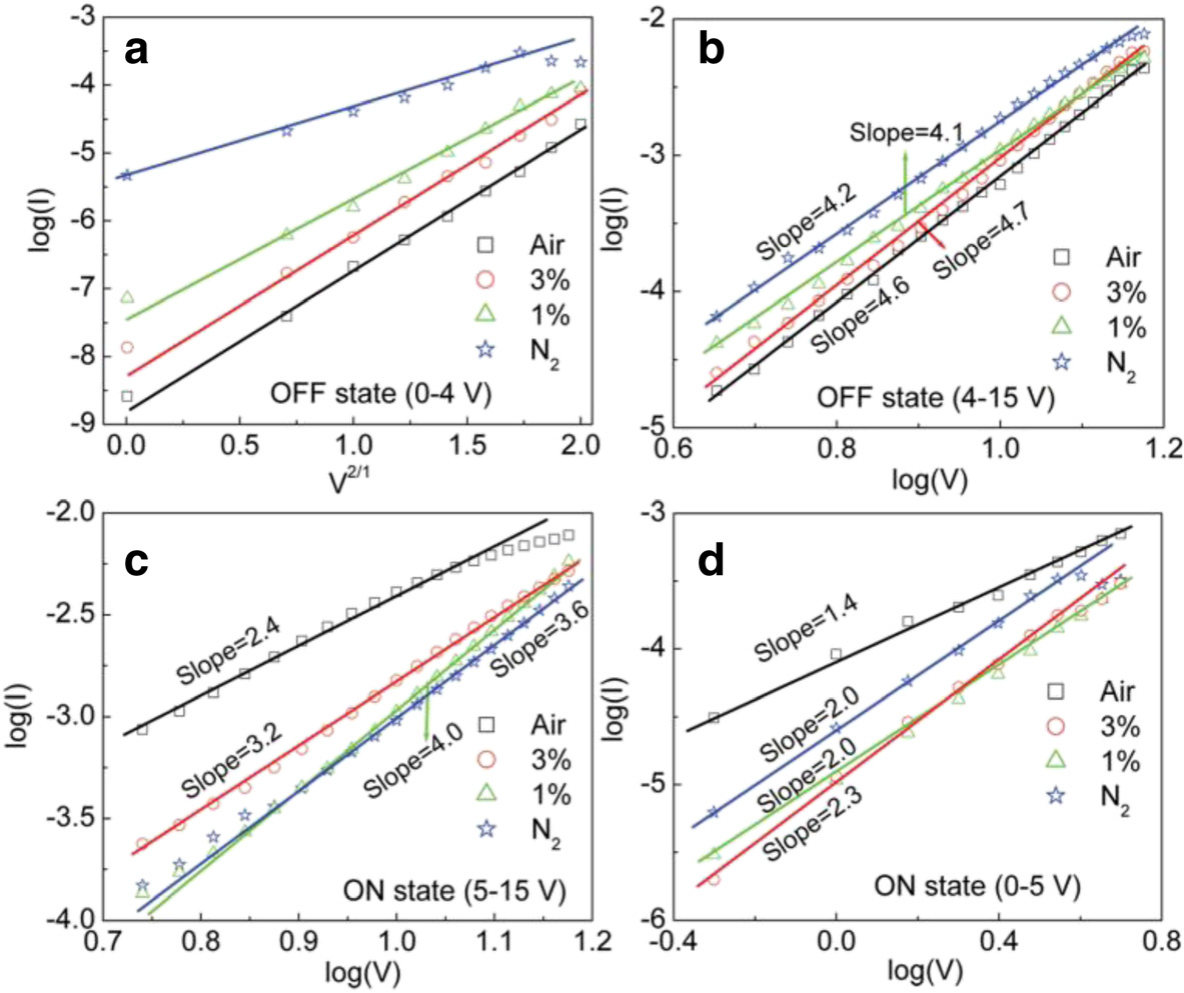
Experimental results (*open scatter*) and the theoretical fitting results (*solid line*) of the *I*-*V* characteristics of the bistable devices measured under different atmospheres in different positive voltage regions. **a** Linear relationship between log *I* versus *V* ^1/2^ in the voltage region of 0–4 V (*OFF state*); linear fitting in a double logarithmic plot in the region of **b** 4–15 V (*OFF state*), **c** 5–15 V (*ON state*), **d** 0–5 V (*ON state*)

In order to get a further understanding of the conducting mechanism of the electrically bistable devices based on hybrid Ag_2_S/PVK nanocomposites measured under different atmospheres, the data fitting of the *I*-*V* characteristics for the devices has been performed by using the following three theoretical models of organic electronics: the thermal emission (TE) model, space-charge-limited-current (SCLC) model, and Ohmic conduction model, which are typically used for studying the carrier transport of organic devices [20–24]. Figure 5a, b shows the experimental *I*-*V* results and the corresponding fitting results for the OFF states in the positive voltage regions. As shown in Fig. 5a, the *I*-*V* characteristics can be well fitted by using the TE model in the voltage region of 0–4 V, and a linear relationship between log *I* and *V*^1/2^ is obtained in the devices measured under different atmospheres [20]. This indicates that the carrier transport at the relatively low voltage region can be dominated by the thermal emission of the charge carrier over the interfacial barrier between the electrode and the active layer. As the sweeping voltage increases from 4 to 15 V, the log *I*-log *V* characteristics of the devices measured under different atmospheres shown in Fig. 5b exhibit a linear relationship with a similar slope in the range from 4.1 to 4.7, which is consistent with a trap-controlled SCLC (TC-SCLC) model (*I*∝*V*^*α*^, *α* > 2) [21, 22]. It should be noted that the slope of the device is measured in the O_2_ concentration of 3 % and air is higher than that of the device measured under other two different atmospheres. This indicates that the number of the carriers injected into the Ag_2_S/PVK layer is increased greatly when the sweeping voltage is higher than a certain voltage, and the electrons can be captured by the traps of the Ag_2_S nanocrystals with an exponential distribution in the forbidden gap, in which a large number of sulfur vacancies formed on the surface of Ag_2_S nanocrystals act as the charge traps [25]. When the devices are measured in the presence of air, the molecular oxygen may react with the sulfur vacancies to form a bridging oxide dimer to act as a potential barrier around the Ag_2_S nanocrystals [26, 27]. Thus, the number of the injected carriers trapped by the barrier may be increased, which is responsible for the high slope of the fitting line of the devices measured in air.

When a reverse sweeping voltage is applied to the device, the conducting states switch from OFF state to ON state, and the corresponding experimental and fitting *I*-*V* results are depicted in Fig. 5c, d. Two different linear fitting regions in a double logarithmic plot are obtained in the sweeping voltage from 15 to 0 V for the ON state. As shown in Fig. 5c, a distinct linear relationship between log *I* and log *V* with a slope of 2.4 is observed in the device measured in the air for the sweeping voltage range from 15 to 5 V, indicating the electrical conduction is dominated by the SCLC process in the voltage region. In contrast, the slope of the device measured under three different atmospheres is in the range of 3.2–4, indicating that the TC-SCLC process dominates the carrier transport of the device in the relatively low O_2_ concentration and N_2_ in this voltage region. The electrical conduction of the device measured in air changes from TC-SCLC to SCLC, suggesting that the number of the injected carriers is decreased as some trapped carriers are released from the Ag_2_S nanocrystals due to the breakdown of the oxide dimer, and some other carriers are still trapped by the dodecanethiol-capped Ag_2_S nanocrystals. Moreover, with the sweeping voltage decreasing from 5 to 0 V, the slope of the linear fitting of the device measured in air is decreased from 2.4 to 1.4 (Fig. 5d), indicating that the carrier transport changes from the TF-SCLC model to Ohmic conduction [23], and this behavior may be attributed to the formation of conduction filament which acts as a resistor with a low resistivity [28, 29]. However, the slope of the fitting line remains in the range of 2.0–2.3 for the device measured in the relatively low O_2_ concentration and N_2_ atmosphere, which suggests that the electrical conduction is governed by the TF-SCLC model in this voltage range. The aforementioned theoretical fitting results indicate that the electrical conduction of the device for the OFF state is mainly governed by the TE model in the low voltage region and TC-SCLC model in the relatively high voltage region when it is measured under different gas atmospheres. After the conduction state switches from OFF state to ON state, the electrical conduction of the device measured in air is very different from that of the device measured in the low O_2_ concentration and N_2_. This indicates that the oxygen concentration has an important effect on the carrier transport of the device for the ON state.

## Conclusions

In summary, an electrically bistable devices based on hybrid Ag_2_S/PVK nanocomposites was fabricated by using a simple solution process technique. The *I*-*V* characteristics of the device measured in different atmospheres with different oxygen concentrations exhibited electrical bistable behaviors, and the current of ON state and OFF state showed a strong dependence on the oxygen concentration. The conducting and carrier transport mechanisms of the device were studied by using different theoretical models of organic electronics, and the electrical conduction of the device measured in air for the ON state was very different from that of the device measured in low oxygen concentrations. The device may have potential applications both in the industry of gas sensor and memory storage due to its high level of sensitivity to the environmental oxygen and two distinctive resistive conducting states.

## References

[1] Song WS, Yang HY, Yoo CH, Yun DY, Kim TW (2012). Memory stabilities and mechanisms of organic bistable devices with a phase-separated poly (methylmethacrylate)/poly (3-hexylthiophene) hybrid layer. Organic Electronics.

[2] Kim TW, Yang Y, Li F, Kwan WL (2012). Electrical memory devices based on inorganic/organic nanocomposites. NPG Asia Mater.

[3] Ouyang J, Chu CW, Szmanda CR, Ma L, Yang Y (2004). Programmable polymer thin film and non-volatile memory device. Nat Mater.

[4] Tang A, Teng F, Qian L, Hou Y, Wang Y (2009). Electrical bistability of copper (I) sulfide nanocrystals blending with a semiconducting polymer. Appl Phys Lett.

[5] Yook K, Jeon SO, Joo CW, Lee JY, Kim SH, Jang J (2009). Organic bistable memory device using MoO3 nanocrystal as a charge trapping center. Org Electron.

[6] Pal K, Zhan B, Mohan MLNM, Schirhagl R, Wang GP (2015). Influence of ZnO nanostructures in liquid crystal interfaces for bistable switching applications. Appl Surf Sci.

[7] Yun DY, Park HM, Kim SW, Kim SW, Kim TW (2014). Enhancement of memory margins for stable organic bistable devices based on graphene-oxide layers due to embedded CuInS2 quantum dots. Carbon.

[8] Yun DY, Song WS, Kim TW, Kim SW, Kim SW (2012). Electrical stabilities and carrier transport mechanisms of flexible organic bistable devices based on CdSe-InP core-shell nanoparticle/polystyrene nanocomposites. Appl Phys Lett.

[9] Au VK, Wu D, Yam VW (2015). Organic memory devices based on a bis-cyclometalated alkynylgold(III) complex. J Am Chem Soc.

[10] Bozano LD, Kean BW, Deline VR, Salem JR, Scott JC (2004). Mechanism for bistability in organic memory elements. Appl Phys Lett.

[11] Son DI, You CH, Jung JH, Kim TW (2010). Carrier transport mechanisms of organic bistable devices fabricated utilizing colloidal ZnO quantum dot-polymethylmethacrylate polymer nanocomposites. Appl Phys Lett.

[12] Ghosh B, Pal AJ (2009). Conductance switching in TiO2 nanorods is a redox-driven process: evidence from photovoltaic parameters. J Phys Chem C.

[13] Gao S, Song C, Chen C, Zeng F, Pan F (2012). Dynamic processes of resistive switching in metallic filament-based organic memory devices. J Phys Chem C.

[14] Lai YS, Tu CH, Kwong DL, Chen JS (2005). Bistable resistance switching of poly(N-vinylcarbazole) films for nonvolatile memory applications. Appl Phys Lett.

[15] Li J, Tang A, Li X, Cao YP, Wang M, Ning Y, Long LF, Lv QP, Lu YZ, Hu YF, Hou YB, Teng F (2014). Negative differential resistance and carrier transport of electrically bistable devices based on poly(*N*-vinylcarbazole)-silver sulfide composites. Nanoscale Res Lett.

[16] Wang M, Wang Y, Tang A, Li X, Hou Y, Teng F (2012). Optical properties and self-assembly of Ag_2_S nanoparticles synthesized by a one-pot method. Mater Lett.

[17] Yun Y, Arul NS, Lee DU, Lee NH, Kim TW (2015). Memory stabilities and mechanisms of organic bistable devices with giant memory margins based on Cu2ZnSnS4nanoparticles/PMMA nanocomposites. Org Electron.

[18] Tseng ZL, Kao PC, Shih MF, Huang HH, Wang JY, Chu SY (2010). Electrical bistability in hybrid ZnO nanorod/polymethylmethacrylate heterostructures. Appl Phys Lett.

[19] Mukherjee B, Mukherjee M (2009). Nonvolatile memory device based on Ag nanoparticle: characteristics improvement. Appl Phys Lett.

[20] Burroughes JH, Jones CA, Friend RH (1998). New semiconductor device physics in polymer diodes and transistors. Nature (London).

[21] Tang AW, Teng F, Hou YB, Wang YS, Tan FR, Qu SC, Wang ZG (2010). Optical properties and electrical bistability of CdS nanoparticles synthesized in dodecanethiol. Appl Phys Lett.

[22] Vincent G, Chantre A, Bois D (1979). Electric field effect on the thermal emission of traps in semiconductor junctions. J Appl Phys.

[23] Kumar V, Jain SC, Kapoor AK, Geens W, Aernauts T, Poortmans J, Mertens R (2002). Carrier transport in conducting polymers with field dependent trap occupancy. J Appl Phys.

[24] Kapoor AK, Jain SC, Poortmans J, Kumar V, Mertens R (2002). Temperature dependence of carrier transport in conducting polymers: similarity to amorphous inorganic semiconductors. J Appl Phys.

[25] Schaub R, Wahlström E, Rønnau A, Lægsgaard E, Stensgaard I, Besenbacher F (2003). Oxygen-mediated diffusion of oxygen vacancies on the TiO2(110) surface. Science.

[26] Singh B, Mehta BR, Varandani D, Narita A, Feng X, Müllen K (2013). Bipolar resistive switching properties of Ti-CuO/(hexafluoro-hexa-peri-hexabenzocoronene)-Cu hybrid interface device: influence of electronic nature of organic layer. J Appl Phys.

[27] Yao Y, You Y, Si W, Wu CQ (2012). Modeling the underlying mechanisms for organic memory devices: tunneling, electron emission, and oxygen adsorbing. Appl Phys Lett.

[28] Son JM, Song WS, Yoo CH, Yun DY, Kim TW (2012). Electrical stabilities and memory mechanisms of organic bistable devices fabricated utilizing a poly(3,4-ethylene-dioxythiophene): poly(styrenesulfonate) layer with a poly(methyl methacrylate) buffer layer. Appl Phys Lett.

[29] Son I, Kim TW, Shim JH, Jung JH, Lee DU, Lee JM, Park WI, Choi WK (2010). Flexible organic bistable devices based on graphene embedded in an insulating poly(methyl methacrylate) polymer layer. Nano Lett.

